# Supplementary material for: Using hypergraphs to quantify importance of sets of diseases by healthcare resource utilisation: A retrospective cohort study
Source: PLoS One. 2023 Dec 15;18(12):e0295300. doi: 10.1371/journal.pone.0295300 (PMC10723667; doi:10.1371/journal.pone.0295300)
Supplement: Attachment — Submitted filename: plosone_referee_response.docx [file pone.0295300.s007.docx]

Dear Dr Wulandari,

Thank you for reviewing our paper “Using hypergraphs to quantify importance of sets of diseases by healthcare resource utilisation: A retrospective cohort study.” We have included the comments from editors and referees below with our responses in *italic font.*

Best wishes,

Jim Rafferty, on behalf of the authors.

ACADEMIC EDITOR:

Please share in the background section to show that this research is essential and has a specificity that distinguishes it from previous research.

Added an explanation in the method section.

Improve the writing of results and deepen the discussion and conclusions.

Take notice of all reviewer suggestions.

When submitting your revision, we need you to address these additional requirements.

1. Please ensure that your manuscript meets PLOS ONE's style requirements, including those for file naming. The PLOS ONE style templates can be found at 
[https://journals.plos.org/plosone/s/file?id=wjVg/PLOSOne_formatting_sample_main_body.pdf](https://eur03.safelinks.protection.outlook.com/?url=https%3A%2F%2Fjournals.plos.org%2Fplosone%2Fs%2Ffile%3Fid%3DwjVg%2FPLOSOne_formatting_sample_main_body.pdf&data=05%7C01%7CJ.M.Rafferty%40Swansea.ac.uk%7Cf27273a2178848b60f2d08dacdcf1f15%7Cbbcab52e9fbe43d6a2f39f66c43df268%7C0%7C0%7C638048586702542263%7CUnknown%7CTWFpbGZsb3d8eyJWIjoiMC4wLjAwMDAiLCJQIjoiV2luMzIiLCJBTiI6Ik1haWwiLCJXVCI6Mn0%3D%7C3000%7C%7C%7C&sdata=ZTuX4s1vbcygjeZA6IS5t1Id487RBmmZTRZC%2FOK3MtI%3D&reserved=0) and 
[https://journals.plos.org/plosone/s/file?id=ba62/PLOSOne_formatting_sample_title_authors_affiliations.pdf](https://eur03.safelinks.protection.outlook.com/?url=https%3A%2F%2Fjournals.plos.org%2Fplosone%2Fs%2Ffile%3Fid%3Dba62%2FPLOSOne_formatting_sample_title_authors_affiliations.pdf&data=05%7C01%7CJ.M.Rafferty%40Swansea.ac.uk%7Cf27273a2178848b60f2d08dacdcf1f15%7Cbbcab52e9fbe43d6a2f39f66c43df268%7C0%7C0%7C638048586702542263%7CUnknown%7CTWFpbGZsb3d8eyJWIjoiMC4wLjAwMDAiLCJQIjoiV2luMzIiLCJBTiI6Ik1haWwiLCJXVCI6Mn0%3D%7C3000%7C%7C%7C&sdata=roqNi752G82Tp0dEeYtI4HqmeSfrOWAz1au20qkgr78%3D&reserved=0)

*We believe that our manuscript meets PLOS ONE style requirements.*

2. Please ensure that you have specified (1) whether consent was informed and (2) what type you obtained (for instance, written or verbal, and if verbal, how it was documented and witnessed). If your study included minors, state whether you obtained consent from parents or guardians. If the need for consent was waived by the ethics committee, please include this information.

*Our study was performed using anonymised routine data made available for research, so informed consent was not required. Our study cohort only included data on people aged 18 or older.*

3. Please could you clarify within the ethics statement whether the 'Information Governance Review Panel of Swansea University' is the same committee as the Research Ethics - Swansea University.

*The information governance review panel of Swansea University (IGRP) is not the same committee as the research ethics – Swansea University committee. The IGRP is a panel that is convened to provide independent guidance and advice on Information Governance policies, procedures and processes for SAIL Databank. Since our study used anonymised routine data, full ethical approval by an ethics committee was not required.*

4. Thank you for stating the following in the Acknowledgments Section of your manuscript:

This work was supported by the Medical Research Council (MRC), grant no. 232
MR/S027750/1. FJ is supported by a MRC/University of Manchester Skills 233
Development Fellowship (grant number MR/R016615). The funders had no role in 234
study design, data collection and analysis, decision to publish, or preparation of the 235
manuscript.

However, funding information should not appear in the Acknowledgments section or other areas of your manuscript. We will only publish funding information present in the Funding Statement section of the online submission form. 
Please remove any funding-related text from the manuscript and let us know how you would like to update your Funding Statement. Currently, your Funding Statement reads as follows:

This work was supported by the Medical Research Council (MRC), grant no. MR/S027750/1. FJ is supported by a MRC/University of Manchester Skills Development Fellowship (grant number MR/R016615). The funders had no role in study design, data collection and analysis, decision to publish, or preparation of the manuscript.

Please include your amended statements within your cover letter; we will change the online submission form on your behalf.

*Thank you for noting this. We have removed the funding section from the acknowledgement. The funding statement does not require any modification.*

5. In your Data Availability statement, you have not specified where the minimal data set underlying the results described in your manuscript can be found. PLOS defines a study's minimal data set as the underlying data used to reach the conclusions drawn in the manuscript and any additional data required to replicate the reported study findings in their entirety. All PLOS journals require that the minimal data set be made fully available. For more information about our data policy, please see [http://journals.plos.org/plosone/s/data-availability](https://eur03.safelinks.protection.outlook.com/?url=http%3A%2F%2Fjournals.plos.org%2Fplosone%2Fs%2Fdata-availability&data=05%7C01%7CJ.M.Rafferty%40Swansea.ac.uk%7Cf27273a2178848b60f2d08dacdcf1f15%7Cbbcab52e9fbe43d6a2f39f66c43df268%7C0%7C0%7C638048586702542263%7CUnknown%7CTWFpbGZsb3d8eyJWIjoiMC4wLjAwMDAiLCJQIjoiV2luMzIiLCJBTiI6Ik1haWwiLCJXVCI6Mn0%3D%7C3000%7C%7C%7C&sdata=2pvlTTqOWbfITy5vOiboTMRk1fHdTl9bkSDBnIsqcDU%3D&reserved=0).

"Upon re-submitting your revised manuscript, please upload your study’s minimal underlying data set as either Supporting Information files or to a stable, public repository and include the relevant URLs, DOIs, or accession numbers within your revised cover letter. For a list of acceptable repositories, please see [http://journals.plos.org/plosone/s/data-availability#loc-recommended-repositories](https://eur03.safelinks.protection.outlook.com/?url=http%3A%2F%2Fjournals.plos.org%2Fplosone%2Fs%2Fdata-availability%23loc-recommended-repositories&data=05%7C01%7CJ.M.Rafferty%40Swansea.ac.uk%7Cf27273a2178848b60f2d08dacdcf1f15%7Cbbcab52e9fbe43d6a2f39f66c43df268%7C0%7C0%7C638048586702542263%7CUnknown%7CTWFpbGZsb3d8eyJWIjoiMC4wLjAwMDAiLCJQIjoiV2luMzIiLCJBTiI6Ik1haWwiLCJXVCI6Mn0%3D%7C3000%7C%7C%7C&sdata=PXwbigS1x6SXz89ZNnI7U3Tq%2FSjeB%2FHDIkiSVOxyn1k%3D&reserved=0). Any potentially identifying patient information must be fully anonymized.

Important: If there are ethical or legal restrictions to sharing your data publicly, please explain these restrictions in detail. Please see our guidelines for more information on what we consider unacceptable restrictions to publicly sharing data: [http://journals.plos.org/plosone/s/data-availability#loc-unacceptable-data-access-restrictions](https://eur03.safelinks.protection.outlook.com/?url=http%3A%2F%2Fjournals.plos.org%2Fplosone%2Fs%2Fdata-availability%23loc-unacceptable-data-access-restrictions&data=05%7C01%7CJ.M.Rafferty%40Swansea.ac.uk%7Cf27273a2178848b60f2d08dacdcf1f15%7Cbbcab52e9fbe43d6a2f39f66c43df268%7C0%7C0%7C638048586702542263%7CUnknown%7CTWFpbGZsb3d8eyJWIjoiMC4wLjAwMDAiLCJQIjoiV2luMzIiLCJBTiI6Ik1haWwiLCJXVCI6Mn0%3D%7C3000%7C%7C%7C&sdata=Ak5Hx0ouU9cJUSCb5beMzOYCFlRfOnXSdVKoHNPMAnM%3D&reserved=0). Note that it is not acceptable for the authors to be the sole named individuals responsible for ensuring data access.

We will update your Data Availability statement to reflect the information you provide in your cover letter.

*This study makes use of anonymised, individual-level data held in the SAIL Databank, a Trusted Research Environment, at Swansea University, Swansea, UK. Due to the nature and level of the data, data are not publicly available but are available to researchers upon application. All proposals to use SAIL data are subject to review by the independent Information Governance Review Panel (IGRP). The IGRP gives careful consideration to each project proposal to ensure appropriate use of SAIL data. If a project is approved, access to the requested data is gained through a privacy-protecting safe haven and remote access system referred to as the SAIL Gateway. SAIL has established an application process to be followed by anyone who would like to access data at: https://www.saildatabank.com/application-process/.*

6. Please include your full ethics statement in the ‘Methods’ section of your manuscript file. In your statement, please include the full name of the IRB or ethics committee who approved or waived your study, as well as whether or not you obtained informed written or verbal consent. If consent was waived for your study, please include this information in your statement as well.

*As noted above, ethical approval was not required for this study. We have added the following remarks to the methods section:*

*“This study was performed using anonymised routine data held in the Secure Anonymised Information Linkage (SAIL) Databank, a Trusted Research Environment for people interacting with healthcare services in Wales, UK. The study was approved by the Information Governance Review Panel under project reference number 0911. Ethical approval was not required nor sought for the study.”*

7. Please include captions for your Supporting Information files at the end of your manuscript, and update any in-text citations to match accordingly. Please see our Supporting Information guidelines for more information: [http://journals.plos.org/plosone/s/supporting-information](https://eur03.safelinks.protection.outlook.com/?url=http%3A%2F%2Fjournals.plos.org%2Fplosone%2Fs%2Fsupporting-information&data=05%7C01%7CJ.M.Rafferty%40Swansea.ac.uk%7Cf27273a2178848b60f2d08dacdcf1f15%7Cbbcab52e9fbe43d6a2f39f66c43df268%7C0%7C0%7C638048586702542263%7CUnknown%7CTWFpbGZsb3d8eyJWIjoiMC4wLjAwMDAiLCJQIjoiV2luMzIiLCJBTiI6Ik1haWwiLCJXVCI6Mn0%3D%7C3000%7C%7C%7C&sdata=nmGosRvZoAWs9osZpZx4CFkRMEF8Yl9ZIESYV%2BsMOG8%3D&reserved=0).

*We have added the supplementary material captions to the end of the manuscript document.*

8. Please review your reference list to ensure that it is complete and correct. If you have cited papers that have been retracted, please include the rationale for doing so in the manuscript text, or remove these references and replace them with relevant current references. Any changes to the reference list should be mentioned in the rebuttal letter that accompanies your revised manuscript. If you need to cite a retracted article, indicate the article’s retracted status in the References list and also include a citation and full reference for the retraction notice.

*We have added several references to the manuscript (detailed in other responses to comments) and believe it to be complete. All the other references are relevant and current to our knowledge.*

Additional Editor Comments:

This post is exciting and well-written. However, here are some areas that need improvement.

Background.
The author explains that the most frequently used approach so far in multi-morbidity investigations is statistical modeling and unsupervised machine learning. Before going into the explanation of hypergraphs, the author should first explain what are the weaknesses of statistical modeling and unsupervised machine learning, so it is necessary to do research with hypergraphs.

*We have added the following paragraph to the background section:*

*“Graphs and hypergraphs have several advantages over other clustering and statistical modelling approaches. Many clustering methods (for example, hierarchical clustering) allow the user to only include each disease in a single cluster, which may obscure interactions where a single disease is an important feature of several disease clusters. Graph based approaches are highly flexible in that one can use a single framework to quantify different aspects of disease interaction and importance (as we will demonstrate in this work). One may change the interpretation of the results of the graph analysis by changing the weighting scheme used to construct the graph. Performing additional analysis with separate models to obtain more information about the most important disease clusters derived from a hypergraph analysis is not required.”*

The author also needs to add examples of the advantages of using hypergraphs based on previous research.

*The use of hypergraphs is a relatively new approach in multimorbidity research and we are only aware of one other study that has done it (Larvin, H., et al. "Systemic Multimorbidity Clusters in People with Periodontitis." Journal of Dental Research (2022): 00220345221098910). We have added this reference.*

Writers should write down the research objectives clearly.

*We have added the following to the end of the introduction:*

*“This aims of this work were*

*1. To demonstrate the utility of hypergraphs in their application to problems of quantifying disease set importance based on healthcare resource use, a metric unrelated to the prevalence of the diseases.*

*2. To find the most important sets of diseases based on two different measures of healthcare resource use, interactions with outpatient services and unplanned interactions with inpatient services.”*

Method
Page 2 line 46, the first sentence is confusing, because the author wrote down the cohort used for this research has been explained before. It will be easier for readers to understand if the writer starts by explaining why they use cohort data.

*We have modified the first sentences of the methods to read:*

*“The cohort used for this study consisted of all people living in Wales, UK on the 1st January 2000 and aged 20 years or older, which was constructed specifically to study multimorbidity in Wales, UK. Please see [26] for a full description of the cohort used.”*

The author also has not explained well how to get cohort data.

*As mentioned above, the data is not publicly available due to its sensitivity. Researchers are free to apply to the information governance review panel to gain access to the data.*

How does the author control the quality of the data?

*A discussion of data quality would be outside the scope of this paper. We direct the referee to the references by R Lyons, J Lyons and Ford which discuss in detail the data management of SAIL and the cohort used.*

There should be a more detailed explanation regarding the utilization of health resources.

*We direct the referee to the section entitled “Hypergraph data” in the methods where there is an extensive description of how healthcare resource use was quantified and used to calculate the hypergraph weights.*

Discussion
The author needs to elaborate on more literature and previous research results so that the discussion is more in-depth and interesting.
Try doing the ending separately.

*We have added the following paragraph and references to the discussion:*

*“Our results are coherent on the relatively small but growing literature on the impacts of multimorbidity. Soley-bori and colleagues carried out a systematic review of the impact of multimorbidity on healthcare costs and utilisation in the UK in 2020, identifying 17 studies (7 on costs and 10 on HRU). Whilst the different studies used different demographic inclusion criteria, grouping of morbidity, and time frames the overall patterns were similar; multimorbidity found to be associated with increased primary care, emergency department and inpatient resources. Similar patterns have been reported from Denmark, India, Catalonia and China, again using different categories and methodologies.”*

*Soley-Bori M, Ashworth M, Bisquera A, Dodhia H, Lynch R, Wang Y, et al. Impact of multimorbidity on healthcare costs and utilisation: a systematic review of the UK literature. British Journal of General Practice. 2021;71(702):e39–e46.*

*Frølich A, Ghith N, Schiøtz M, Jacobsen R, Stockmarr A. Multimorbidity, healthcare utilization and socioeconomic status: a register-based study in Denmark. PloS one. 2019;14(8):e0214183.*

*Pati S, Swain S, Knottnerus JA, Metsemakers JF, van den Akker M. Magnitude and determinants of multimorbidity and health care utilization among patients attending public versus private primary care: a cross-sectional study from Odisha, India. International journal for equity in health. 2020;19(1):1–12.*

*Monterde D, Vela E, Cl`eries M, Garcia-Eroles L, Roca J, P ́erez-Sust P. Multimorbidity as a predictor of health service utilization in primary care: a registry-based study of the Catalan population. BMC family practice. 2020;21(1):1–9.*

*Zhao Y, Atun R, Oldenburg B, McPake B, Tang S, Mercer SW, et al. Physical multimorbidity, health service use, and catastrophic health expenditure by socioeconomic groups in China: an analysis of population-based panel data. The Lancet Global Health. 2020;8(6):e840–e849.*

[Note: HTML markup is below. Please do not edit.]

Reviewers' comments:

Reviewer's Responses to Questions

**Comments to the Author**

1. Is the manuscript technically sound, and do the data support the conclusions?

The manuscript must describe a technically sound piece of scientific research with data that supports the conclusions. Experiments must have been conducted rigorously, with appropriate controls, replication, and sample sizes. The conclusions must be drawn appropriately based on the data presented.

Reviewer #1: Yes

2. Has the statistical analysis been performed appropriately and rigorously?

Reviewer #1: Yes

3. Have the authors made all data underlying the findings in their manuscript fully available?

The [PLOS Data policy](https://eur03.safelinks.protection.outlook.com/?url=http%3A%2F%2Fwww.plosone.org%2Fstatic%2Fpolicies.action%23sharing&data=05%7C01%7CJ.M.Rafferty%40Swansea.ac.uk%7Cf27273a2178848b60f2d08dacdcf1f15%7Cbbcab52e9fbe43d6a2f39f66c43df268%7C0%7C0%7C638048586702542263%7CUnknown%7CTWFpbGZsb3d8eyJWIjoiMC4wLjAwMDAiLCJQIjoiV2luMzIiLCJBTiI6Ik1haWwiLCJXVCI6Mn0%3D%7C3000%7C%7C%7C&sdata=MsL8o5l6vSTk2O5onpZwobDCNCOMRnwV%2BWv5a%2B7Uwtg%3D&reserved=0) requires authors to make all data underlying the findings described in their manuscript fully available without restriction, with rare exception (please refer to the Data Availability Statement in the manuscript PDF file). The data should be provided as part of the manuscript or its supporting information, or deposited to a public repository. For example, in addition to summary statistics, the data points behind means, medians and variance measures should be available. If there are restrictions on publicly sharing data—e.g. participant privacy or use of data from a third party—those must be specified.

Reviewer #1: No

4. Is the manuscript presented in an intelligible fashion and written in standard English?

PLOS ONE does not copyedit accepted manuscripts, so the language in submitted articles must be clear, correct, and unambiguous. Any typographical or grammatical errors should be corrected at revision, so please note any specific errors here.

Reviewer #1: Yes

5. Review Comments to the Author

Please use the space provided to explain your answers to the questions above. You may also include additional comments for the author, including concerns about dual publication, research ethics, or publication ethics. (Please upload your review as an attachment if it exceeds 20,000 characters)

Reviewer #1: The authors clearly showed different approaches that have been used to analysed multi-morbidity outcome data citing examples. The further explain how different and noble their methods can be also adopted to analyse a different dimension of such multi-morbid outcome data.

For table 1 it will be best if the diseases are sorted either in ascending or descending order based on the percentages. The current arrangement does not seem to follow any reason order.

*Thank you for this comment. We agree and have reordered the table in descending order of percentage diagnosed.*

Given that hypertension seems to be the most prevailing condition, perhaps, the authors should also consider in future work a similar approach where they focus attention on multimorbidity among patients with hypertension. This will highlight the most important co-morbid conditions related to hypertension

*Thank you for this comment. Further work in this area would certainly be of interest. We have added the following remark to the discussion:*

*“For future work it may be of interest to perform this analysis in cohorts of people with specific, common diseases to understand the common sets of comorbidities and HRU in those subcohorts. From this study, the most interesting populations to explore in studies of this type would be people with hypertension, diabetes or depression.”*

6. PLOS authors have the option to publish the peer review history of their article ([what does this mean?](https://eur03.safelinks.protection.outlook.com/?url=https%3A%2F%2Fjournals.plos.org%2Fplosone%2Fs%2Feditorial-and-peer-review-process%23loc-peer-review-history&data=05%7C01%7CJ.M.Rafferty%40Swansea.ac.uk%7Cf27273a2178848b60f2d08dacdcf1f15%7Cbbcab52e9fbe43d6a2f39f66c43df268%7C0%7C0%7C638048586702542263%7CUnknown%7CTWFpbGZsb3d8eyJWIjoiMC4wLjAwMDAiLCJQIjoiV2luMzIiLCJBTiI6Ik1haWwiLCJXVCI6Mn0%3D%7C3000%7C%7C%7C&sdata=RwJodbh5U8950GKrv5b3ZeY5cT2VtvjUIYXgiXNdq70%3D&reserved=0)). If published, this will include your full peer review and any attached files.


If you choose “no”, your identity will remain anonymous but your review may still be made public.


**Do you want your identity to be public for this peer review?** For information about this choice, including consent withdrawal, please see our [Privacy Policy](https://eur03.safelinks.protection.outlook.com/?url=https%3A%2F%2Fwww.plos.org%2Fprivacy-policy&data=05%7C01%7CJ.M.Rafferty%40Swansea.ac.uk%7Cf27273a2178848b60f2d08dacdcf1f15%7Cbbcab52e9fbe43d6a2f39f66c43df268%7C0%7C0%7C638048586702542263%7CUnknown%7CTWFpbGZsb3d8eyJWIjoiMC4wLjAwMDAiLCJQIjoiV2luMzIiLCJBTiI6Ik1haWwiLCJXVCI6Mn0%3D%7C3000%7C%7C%7C&sdata=xNc%2Bt4BL0i%2BD7RVCP0nGr8WMcmsbZDen6%2By2CLlMibA%3D&reserved=0).

Reviewer #1: No
