# Supplementary material for: Using hypergraphs to quantify importance of sets of diseases by healthcare resource utilisation: A retrospective cohort study
Source: PLoS One. 2023 Dec 15;18(12):e0295300. doi: 10.1371/journal.pone.0295300 (PMC10723667; doi:10.1371/journal.pone.0295300)
Supplement: Attachment — Submitted filename: plosone_referee_response_2_final.docx [file pone.0295300.s008.docx]

Dear Dr Wulandari,

Thank you for providing a follow up review of our paper “Using hypergraphs to quantify importance of sets of diseases by healthcare resource utilisation: A retrospective cohort study.” We have included the comments from the referee below with our responses in *italic* *font* and changes to the manuscript in red text.

Best wishes,

Jim Rafferty, on behalf of the authors.

Journal Requirements:

Please review your reference list to ensure that it is complete and correct. If you have cited papers that have been retracted, please include the rationale for doing so in the manuscript text, or remove these references and replace them with relevant current references. Any changes to the reference list should be mentioned in the rebuttal letter that accompanies your revised manuscript. If you need to cite a retracted article, indicate the article’s retracted status in the References list and also include a citation and full reference for the retraction notice.

*We believe our reference list is complete and that all cited references are published in reputable sources.*

Reviewers' comments:

Reviewer #2: This paper deals with an important problem. How do we deal with the thorny problem of multimorbidity - it is a high dimensional problem. They present a dimension reduction technique, the use of hypergraphs. The data used is suitable for addressing this question. It is good that code is provided.

However, the problem is that, after reading the paper, I am none the wiser as to the role of hypergraphs. I can't interpret figure 2 and am not really sure what the algorithm is doing or what the significance of that is. I cannot bridge the gap between the technical explanation and what is presented. The method is so unfamiliar, and it is the method that is the novel thing here, that I think the authors need to take us through it, almost in the form of a tutorial. I appreciate that this is challenging, but I think we need much more help as readers to understand what is being proposed. Could the authors bring in simulated examples, or examples from other fields?

*Thank you for this comment. We have extensively rephrased the background section of the paper which we hope improves the clarity of the methods. Our previous paper on the use of hypergraphs in multimorbidity research explained in detail how hypergraphs are applied to the problem of multimorbidity and interpreted, which we hope will provide the requested tutorial on the analysis method so we direct the referee to that paper.*

*Rafferty J et al. Ranking sets of morbidities using Hypergraph centrality. Journal of Biomedical Informatics. 2021;122:103916.*

*The final paragraph of the background section now reads:*

*“Research originally in [24] and subsequently performed in [25] demonstrated that hypergraphs were useful in the analysis of multi-morbidity. The advantage of a hypergraph over a binary graph is that hypergraphs can quantify the effect of interactions between any number of diseases (as opposed to just two in a binary graph). This is because a hypergraph is a generalisation of a binary graph where edges can connect any number of nodes. This generalisation leads to a symmetry between nodes and edges that does not exist for binary graphs. Diseases, represented by nodes and sets of diseases, represented by edges can be treated on an equal footing in a hypergraph which allows one to quantitatively estimate the importance of each single disease, disease set and single diseases and sets of diseases together. A detailed account of how hypergraphs are applied to multimorbidity data is given in [24].”*

1. I am afraid that I cannot follow what is meant in the background in the paragraph starting "Research". For example what does "This symmetry allows one to construct the dual hypergraph" mean? This approach is much less familiar to readers than is regression modelling. I think we need some examples and we need to get some kind of intuition or feel for what the algorithm is doing and what it means.

*Apologies for the lack of clarity. We hope the modifications to the background section of the manuscript above improve the work.*

2. I don't think the term "important" is helpful here either when discussing prevalence of HRUs or their combination. Could it instead talk about the most common and most costly? For their combination some other term would be useful. Maybe most "prominent"?

*Thank you. We have considered this carefully. We chose to use the word “important” as a proxy for “most central.” In our view, “importance” is the best choice of word to use because the concept of centrality does indeed provide an estimate of how influential or important a node or edge is to the graph. We believe “important” is a better choice than “prominent” or “prevalent,” because these words imply the disease or set of diseases is common which is not always the case, and “important” is commonly used as a general term for centrality in the context of graph theory. We do agree that “important” is a somewhat loaded word, so we have added some additional explanation of the use of the word in the methods section.*

*“The eigenvector centrality of the nodes and edges of a hypergraph give a direct measure of the importance of each to the graph as a whole and as such are interpreted as the importances of the diseases and sets of diseases.”*

3. The text on Figure 2 is too small. More importantly, we need some text to talk us through the figure to help explain it.

*Thank you for highlighting this. We have increased the size of the axis labels on the figures throughout the manuscript.*

*In addition, we have modified paragraph 4 of the results to read:*

*“When combined to investigate sets of diseases that were important for both prevalence and one of the HRU hypergraphs, we found the most important set of diseases for overlap coefficient combined with unplanned inpatient HRU was arrhythmia, heart failure and hypertension while for overlap coefficient combined with outpatient HRU the most important set was diabetes and hypertension. See figure 2 for a plot of the overlap coefficient hypergraph centrality against the HRU weighted hypergraph centrality and supplemental material for tables of the 100 most important sets. In figure 2, each point represents a set of diseases, and the distance from the origin of each point represents the combined importance of the set of diseases. The colour of the points represents the number of diseases in the set. It is evident that larger sets of diseases typically have larger HRU centrality values, while smaller sets of diseases typically have larger overlap coefficient centrality values.”*

________________________________________

7. PLOS authors have the option to publish the peer review history of their article (what does this mean?). If published, this will include your full peer review and any attached files.

If you choose “no”, your identity will remain anonymous but your review may still be made public.

Do you want your identity to be public for this peer review? For information about this choice, including consent withdrawal, please see our Privacy Policy.

Reviewer #2: Yes: David A McAllister
