# Supplementary material for: Using hypergraphs to quantify importance of sets of diseases by healthcare resource utilisation: A retrospective cohort study
Source: PLoS One. 2023 Dec 15;18(12):e0295300. doi: 10.1371/journal.pone.0295300 (PMC10723667; doi:10.1371/journal.pone.0295300)
Supplement: Attachment — Submitted filename: plosone_referee_response_3.docx [file pone.0295300.s009.docx]

Dear Dr Wulandari,

Thank you for providing a third review of our paper “Using hypergraphs to quantify importance of sets of diseases by healthcare resource utilisation: A retrospective cohort study.” We have included the comments from the referee below with our responses in *italic font* and changes to the manuscript in red text.

Best wishes,

Jim Rafferty, on behalf of the authors.

**Referee Comments**

Thanks for highlighting the Rafferty et al paper (ref 24) using hypergraphs for multimorbidity. The explanations in that were great. It also gave a nice intuition for the concept of the centrality metric and the example of Google's algorithm was very helpful. It would be good to make that paper more prominent in the background.

I agree that in the light of the Rafferty et al paper it is not necessary to write a tutorial-type paper here. However, I think this paper still needs a bit more to give the reader an intuitive understand both networks and centrality as a measure. Something analogous to the papers that reported network meta-analyses before this became a common approach.

I think the paper needs something like the following in the introduction (not my area so the following likely to be wrong, but to give the tone):-

- Describing and summarising multimorbidity is challenging because ...

- approaches that have been used include simple counts, weighted counts, clustering algorithms, and multi-state models (as per current intro)

- Network based approaches are promising because in describing sets of conditions, they allow us to account for the prevalence of individual conditions as well as their relations to other conditions, with varying degrees of commonness. We used a network approach in a previous original article and tutorial paper (24)

- we used hypergraphs for that article rather than simpler binary graphs because XXX and generated summary measures of XXX called centrality. Centrality measures are good because XXX [some intuitive description in the intro]

- Prevalence, is only one way to calculate ... it is possible to weight by any continuous characteristic, and doing so can give different insights into conditions and their relations.

- We now do so for healthcare resource utilisation, which like prevalence is a very important ...

As an aside, I think having a background and an introduction is overkill. It would be better to collapse these into a single section.

*We would like to thank the referee for this and previous comments on the manuscript. We agree and have combined the introduction and background sections and rewritten them using the suggested structure. The modified paragraphs of the introduction are reproduced below for reference.*

*“The use of statistical modelling and unsupervised machine learning techniques to find clusters of coincident diseases and understand multi-morbidity has been thoroughly explored in the literature recently [16]. Several groups observed that network analysis could be used to describe a system of diseases and the interactions between pairs of disease (see for example [17–23]).*

*Network based approaches utilising mathematical structures called graphs and hypergraphs have several advantages over other clustering and statistical modelling approaches. Many clustering methods (for example, hierarchical clustering) allow the user to only include each disease in a single cluster, which may obscure interactions where a single disease is an important feature of several disease clusters. Approaches utilising weighted graphs are useful since they allow us to account for the prevalence of individual conditions while also accounting for the number of people that have different combinations of diseases (which can be thought of as a measure of ‘prevalence’ of sets of diseases).*

*We used a hypergraph rather than a simpler binary graph for this work because hypergraphs can quantify the effect of interactions between any number of diseases (as opposed to just two in a binary graph). The calculation of a quantity from the graph called centrality allows one to quantitatively estimate the connectedness of nodes within the graph. Nodes with a high centrality are strongly connected to other nodes, which for a graph where nodes represent diseases and the edge weights represent the number of people that have all diseases connected to the edge, represents the frequency at which a single disease features in sets of diseases (and hence, it’s importance to multi-morbidity). We used a network approach and a hypergraph in a previous original article and tutorial paper [24]. Subsequently, work using hypergraphs has been performed by others [25].*

*The measure of prevalence of sets of diseases is only one choice of weighting scheme. Any measure related to the sets of diseases can be used to weight the hypergraph, which reflects the flexibility of the approach. In this study we have chosen to use healthcare resource utilisation as the quantity with which we will weight the graph, since healthcare resource use is an important factor to understand for healthcare delivery planning purposes, and is likely to be highly correlated with negative patient outcomes.*

*This aims of this work were
1. To demonstrate the utility of hypergraphs in their application to problems of quantifying disease set importance based on healthcare resource use, a metric unrelated to the prevalence of the diseases.
2. To find the most important sets of diseases based on two different measures of healthcare resource use, interactions with outpatient services and unplanned interactions with inpatient services, and a similar measure of prevalence to that used in previous work [24] making a total of three weightings for consideration.”*
